# Ferroptosis: a double-edged sword that enhances radiation sensitivity and facilitates radiation-induced injury in tumors

**DOI:** 10.3389/fimmu.2025.1591172

**Published:** 2025-07-10

**Authors:** Yang Liu, Jianhao Zhan, Jisheng Wang, Xiaoping Zeng, Shanshan Liu, Le Huang, Liyan Niu, Chengpeng Sun, Zijun Ding, Yan Xing, Zhengyu Zhou, Xiaoying Li, Qing Li, Hongmei Wang

**Affiliations:** ^1^ Department of Oncology, The Second Affiliated Hospital of Nanchang University, Nanchang, Jiangxi, China; ^2^ The First School of Clinical Medicine of Nanchang University, Nanchang University, Nanchang, Jiangxi, China; ^3^ HuanKui Academy, Nanchang University, Nanchang, Jiangxi, China; ^4^ Medical College, Jinhua University of Vocational Technology, Jinhua, Zhejiang, China; ^5^ School of Basic Medical Sciences, Nanchang University, Nanchang, Jiangxi, China

**Keywords:** radiotherapy, ferroptosis, radiotherapy sensitivity, radiation-induced injury, pharmacotherapy

## Abstract

Cell death is a crucial mechanism by which radiotherapy eliminates tumor cells. Ferroptosis, characterized by intracellular iron overload and lipid peroxidation, represents a distinct form of programmed cell death. Recent research has demonstrated that numerous malignant tumors exhibit high sensitivity to ferroptosis. Therefore, the induction of ferroptosis in tumor cells has emerged as a promising approach to overcome apoptosis resistance and increase sensitivity to radiotherapy. In this review, we aim to shed light on ferroptosis and its dual roles in both enhancing radiation sensitivity in tumor cells and facilitating radiation-induced injury. Then we discussed the contradiction of ferroptosis between radiation sensitivity and radiation-induced injury, providing valuable insights and directions for the advancement of clinical tumor radiotherapy.

## 1 Introduction

Radiotherapy is currently the main method of clinical treatment for various malignant tumors. Its mechanism involves the use of high-energy ionizing radiation (IR) to induce DNA fragmentation in tumor cells and subsequent initiation of various intracellular signal transduction pathways, including oxidative stress, the inflammatory response, and apoptosis (1). Nevertheless, tumor cells can develop resistance to radiotherapy through various mechanisms, such as DNA damage repair, cell cycle arrest, inhibition of apoptosis, hypoxia, and immune suppression, which seriously affects the efficacy of radiotherapy (2).

Therefore, strategies are needed to effectively kill radioresistant tumor cells and improve their sensitivity to radiotherapy. Researchers have continually searched for other methods to completely kill tumor cells, such as restoring the ability of tumor cells to undergo apoptosis or inducing other forms of cell death in tumor cells. In 2012, Dixon first proposed the concept of ferroptosis (3). In contrast to apoptosis, ferroptosis is an iron-dependent cell death mechanism caused by lipid peroxidation. Increasing evidence indicates that ferroptosis plays an important role in cancer (4). Further research revealed that many refractory tumors are more sensitive to ferroptosis than apoptosis (5). Since apoptosis is dysfunctional in tumor cells (6), inducing ferroptosis may compensate for defects caused by apoptosis resistance and increase radiation sensitivity. This provides new prospects for clinical radiotherapy.

However, the cytotoxic effects of IR are not specific. In the irradiated area, in addition to tumor cells, there are also normal tissue cells that undergo oxidative stress and inflammatory reactions upon radiation exposure. The resulting tissue and organ damage is known as radiation-induced injury (7). Radiation-induced injury significantly affects the survival time and quality of life of cancer patients undergoing radiotherapy. Recent studies have shown that in addition to traditional oxidative stress and inflammatory pathways, ferroptosis appears to play a crucial role in radiation-induced injury (8). Ferroptosis, a distinct form of cell death induced by radiotherapy, plays a significant role in killing tumor cells and enhancing radiotherapy sensitivity. However, if radiation-induced ferroptosis occurs in normal cells, it can cause damage and death. Inhibiting ferroptosis has become an important strategy for the prevention and treatment of radiation-induced injury in clinical practice.

In this review, we focused on ferroptosis and summarized the relationship between ferroptosis induction, which enhances radiotherapy sensitivity, and ferroptosis inhibition, which mitigates radiation-induced injury. We thoroughly discussed the bidirectional role of ferroptosis in radiotherapy, providing new strategies and insights for improving tumor radiotherapy sensitivity and reducing radiation-induced injury in clinical practice.

## 2 The mechanism and regulation pathways of ferroptosis

Ferroptosis is an iron-dependent form of programmed cell death, characterized by uncontrolled accumulation of lipid peroxidation, leading to disruption of cell membrane structure and cell death (3). This process involves complex interactions among iron metabolism, lipid metabolism, antioxidant systems, and multiple signaling pathways. The following is a comprehensive analysis of the molecular mechanisms of ferroptosis from multiple dimensions.

### 2.1 Core driving role of iron metabolism

As a core driver of ferroptosis, iron deeply participates in the cascade reaction of ferroptosis through its unique redox activity and metabolic network (9). At the level of iron homeostasis regulation, cells achieve efficient iron uptake through the classical endocytic pathway mediated by transferrin receptor 1 (TFR1) (10): he complex formed by plasma transferrin (TF) and Fe³^+^ is endocytosed, converted to Fe²^+^ by reductases such as STEAP3 in acidic endosomes, and then transported to the cytoplasm through DMT1 to form a labile iron pool (LIP). This dynamic reservoir is not only the core hub of cellular iron metabolism but also a key source of triggering oxidative stress (11). Iron regulatory proteins IRP1/IRP2 dynamically regulate the expression of iron metabolism-related genes by sensing changes in intracellular iron concentration and specifically binding to the iron response elements (IREs) of target gene mRNAs. In iron-deficient states, IRPs enhance iron uptake efficiency and block iron chelation and storage by stabilizing TFR1 mRNA and inhibiting ferritin translation, thus maintaining the active state of LIP (12). Under ferroptosis-inducing conditions, the heteropolymer composed of ferritin heavy chain (FTH1) and light chain (FTL) is selectively degraded through the autophagy-lysosome pathway, which is driven by the cargo recognition mechanism mediated by NCOA4. This leads to the abnormal release of a large amount of Fe²^+^ from the ferritin nanocage into the cytoplasm, causing the LIP concentration to exceed the physiological threshold. Overloaded Fe²^+^ reacts with hydrogen peroxide (H_2_O_2_) through the Fenton reaction to generate highly reactive hydroxyl radicals (·OH) (13),, which directly attack the phospholipid bilayer rich in polyunsaturated fatty acids, triggering a chain reaction of lipid peroxidation and forming lipid peroxides (LPO) with cell membrane penetrability, ultimately leading to the disintegration of cell membrane integrity. Meanwhile, the systemic iron homeostasis regulator hepcidin inhibits iron recycling in macrophages and iron efflux in intestinal epithelial cells by binding to and internalizing ferroportin (FPN), forming an “iron lock” effect and further amplifying the positive feedback loop of intracellular iron accumulation and oxidative damage (14).This synergistic effect between iron metabolism imbalance and oxidative stress makes iron ions not only a catalyst for lipid peroxidation but also a molecular bridge connecting the ferroptosis signaling network with cellular redox collapse.

### 2.2 Cascade amplification mechanism of lipid peroxidation

As the core execution mechanism of ferroptosis, lipid peroxidation begins with the specific enrichment and oxidative modification of polyunsaturated fatty acids (PUFAs) in membrane phospholipids. In the initiation stage of ferroptosis, long-chain fatty acid CoA ligase 4 (ACSL4) catalyzes the binding of PUFAs (such as arachidonic acid AA and adrenic acid AdA) to CoA via thioester bonds to form activated fatty acyl-CoA derivatives (15), which are then precisely integrated into the sn-2 site of phosphatidylethanolamine (PE) by lysophosphatidylcholine acyltransferase 3 (LPCAT3) to generate oxidation-sensitive phospholipid molecules such as AA-PE or AdA-PE. These double bond-rich lipid components significantly increase the susceptibility of the cell membrane to oxidative attack by changing the topological structure of membrane phospholipids (16). Members of the lipoxygenase (LOXs) family (such as ALOX15 and ALOXE3) and cytochrome P450 oxidoreductase (POR) together form an oxidative engine: LOXs directly catalyze the molecular oxygen insertion reaction at the double bond region of PUFAs through their non-heme iron active centers to form lipid hydroperoxides (L-OOH) (17), while POR reduces oxidized iron (Fe³^+^) to catalytically active Fe²^+^ through the NADPH-dependent electron transport system, continuously driving the Fenton reaction and accelerating the generation of hydroxyl radicals (·OH), thus forming a self-reinforcing cycle of lipid peroxidation and ROS production (18). Once the antioxidant system fails, L-OOH cannot be reduced to harmless lipid alcohols (L-OH), leading to the abnormal accumulation of lipid peroxides in the local microdomain of the membrane and triggering a radical-mediated chain propagation reaction. The lipid alkoxyl radicals (LO·) and peroxyl radicals (LOO·) generated by the decomposition of L-OOH initiate exponentially amplified oxidative fission by abstracting hydrogen atoms from adjacent lipid molecules, causing lipid peroxidation damage to spread rapidly from the initial site to the entire membrane system (19).The damaged membrane phospholipids lose membrane asymmetry due to the reconstruction of polar head groups, leading to the disintegration of the bilayer structure and ultimately causing plasma membrane rupture and organelle dysfunction. This positive feedback network constructed by enzymatic oxidation, radical diffusion, and iron-dependent redox cycling makes lipid peroxidation irreversibly push cells toward ferroptosis once it exceeds the critical threshold of the antioxidant system.

### 2.3 Dual defense network of the antioxidant system

Cells have constructed a dynamic protective system against ferroptosis, which consists of an enzymatic defense system with the glutathione (GSH)-glutathione peroxidase 4 (GPX4) axis as the core and a multi-layered antioxidant network independent of GPX4. As the core antagonist of lipid peroxidation, GPX4 converts toxic lipid hydroperoxides (L-OOH) into inert lipid alcohols (L-OH) by consuming the reducing power provided by GSH, effectively blocking the propagation of radical chain reactions (20). The integrity of its function depends on the continuous supply of intracellular GSH, which is precisely regulated by the cystine-glutamate antiporter (system Xc^-^) (21). This transporter exchanges extracellular cystine with intracellular glutamate at a 1:1 ratio, and the ingested cystine is converted to cysteine by glutathione reductase, which is then used to generate GSH through the cascade reactions of γ-glutamylcysteine synthetase (GCL) and glutathione synthetase (GSS) (22). When ferroptosis inducers (such as erastin) block cystine uptake by inhibiting system Xc^-^ (23), or small molecules like RSL3 covalently modify the active site selenocysteine residue of GPX4 (24), the disruption of the GSH synthesis pathway or the loss of GPX4 function will lead to the irreversible accumulation of L-OOH in membrane phospholipids, ultimately triggering a lipid peroxidation storm. It is worth noting that cells have evolved GPX4-independent compensatory mechanisms to maintain redox homeostasis. ferroptosis suppressor protein 1 (FSP1) reduces ubiquinone (CoQ10) to ubiquinol (CoQ10H2) through its NAD(P)H-dependent oxidoreductase activity, and the latter, as a lipid-soluble antioxidant, directly captures lipid radicals, forming a new defense axis parallel and independent of GPX4 (25, 26). Meanwhile, GTP cyclohydrolase 1 (GCH1) enhances the antioxidant capacity of membrane phospholipids by regulating the biosynthesis of tetrahydrobiopterin (BH4), and its mechanism may involve the physical protection of BH4 against lipid double bonds or the reduction of superoxide production by regulating nitric oxide synthase activity (27). The synergistic effect of these enzymatic and non-enzymatic defense mechanisms not only constructs a three-dimensional protective network covering the cytoplasm and membrane system in space but also forms a multi-level response from early radical scavenging to late membrane repair in the temporal dimension, enabling cells to dynamically adjust their antioxidant strategies under different stress conditions. The hierarchical collapse of this defense system is a key turning point for the irreversible execution of ferroptosis.

### 2.4 The critical role of mitochondria in ferroptosis

As a core regulatory hub of ferroptosis, mitochondrial dynamic structural and functional disorders run through the entire process of ferroptosis (28). In the initiation stage of ferroptosis, mitochondria first exhibit characteristic morphological remodeling, manifested as mitochondrial matrix shrinkage and abnormal compression of the distance between the outer and inner membranes, leading to a significant reduction in mitochondrial volume and increased membrane density. This structural collapse directly weakens the normal formation and maintenance of mitochondrial cristae, causing the densely folded cristae structure to gradually disintegrate or even disappear (28). Along with the physical changes in the mitochondrial membrane structure, the continuous dissipation of the transmembrane potential (ΔΨm) leads to the disorder of the electron transport chain (ETC), causing abnormal electron leakage from respiratory chain complexes I and II, resulting in a burst of ROS production and forming a vicious oxidative stress cycle (29). It is worth noting that the unique iron metabolism homeostasis regulation mechanism in mitochondria is significantly imbalanced during this process. The disorder of the iron-sulfur cluster (Fe-S) biosynthesis pathway not only weakens the activity of mitochondrial iron chaperone proteins but also leads to the abnormal accumulation of free iron in the mitochondrial matrix (30, 31). This iron-overloaded state exacerbates the generation of hydroxyl radicals through the Fenton reaction (32). At the same time, the polyunsaturated fatty acid phospholipids (PUFA-PL) rich in the mitochondrial membrane phospholipid bilayer constitute unique oxidative vulnerable sites (15) Under the catalysis of iron-dependent lipoxygenase (LOX), these long-chain PUFAs undergo specific peroxidation modification to form cytotoxic lipid peroxides (LPO). The topological characteristics of the mitochondrial membrane structure make these lipid peroxides more likely to form transmembrane gradients locally, ultimately leading to irreversible damage to membrane integrity (33). It is worth exploring that the dynamic opening of the mitochondrial membrane permeability transition pore (mPTP) may play a “molecular switch” role in this process. Its continuous opening not only leads to mitochondrial matrix swelling and rupture of the inner and outer membranes but also may accelerate the ferroptosis process by releasing pro-apoptotic factors and activating downstream death signaling pathways (34). However, the correlation and molecular mechanism between mPTP opening and ferroptosis effectors still need further investigation.

### 2.5 The dynamic regulatory network of ferroptosis signaling pathways

#### 2.5.1 Oxidative stress response pathway

Nrf2, as the central hub of antioxidant defense, binds to Keap1 and is continuously degraded in a resting state (35).The increase in reactive oxygen species induced by ferroptosis promotes a conformational change in Keap1, releasing Nrf2 into the nucleus to initiate the transcription of genes such as HO-1 and NQO1, thereby enhancing the cell’s anti-peroxidation ability by scavenging free radicals and chelating iron ions (35). In contrast, p53 drives ferroptosis through a dual pathway (36): one is to directly inhibit the expression of the SLC7A11 gene, block cystine uptake mediated by system Xc^-^, leading to the interruption of GSH synthesis and the inactivation of GPX4 (37); the other is to activate SAT1 to promote polyamine metabolism and accelerate the lipoxygenase-dependent lipid peroxidation process (38).It is worth noting that post-translational modifications (such as acetylation) or subtype-selective splicing of p53 can dynamically regulate its pro-death activity, revealing its complex regulatory hierarchy in the oxidative stress response (39).

#### 2.5.2 Energy metabolism pathway

In the metabolic regulatory network of ferroptosis, the dynamic balance of the energy metabolism pathway profoundly affects the process of lipid peroxidation. As a core energy sensor, AMPK, when activated by sensing changes in the AMP/ATP ratio, phosphorylates acetyl-CoA carboxylase 1 (ACC1) to inhibit its catalytic activity, block the synthesis of malonyl-CoA, thereby reducing *de novo* fatty acid synthesis and limiting the biosynthesis pool of PUFAs, a key substrate for lipid peroxidation, thereby reducing the risk of oxidative stress (40). LKB1-AMPK signaling axis leads to the dephosphorylation and activation of ACC1, promoting abnormal increases in fatty acid synthesis and significantly enhancing cell sensitivity to ferroptosis (40). Interestingly, the energy stress state exhibits bidirectional regulation: glucose deprivation exerts a protective effect by activating the AMPK-ACC1 axis to inhibit lipid accumulation, while glutamine metabolism enhances the activity of the electron transport chain through the mitochondrial tricarboxylic acid cycle (TCA), promoting the burst of mitochondrial ROS that is difficult to clear by superoxide dismutase (SOD), forming a pro-ferroptotic microenvironment. This metabolic substrate-specific regulatory mechanism suggests that cells can dynamically adjust the ferroptosis threshold by reprogramming the carbon source utilization pattern (such as switching from glucose metabolism to glutamine metabolism), providing a theoretical basis for intervening in ferroptosis by targeting energy metabolism (41).

#### 2.5.3 Immune regulation pathway

The interactive regulation between ferroptosis and antitumor immunity is very complex. On the one hand, ferroptotic tumor cells release damage-associated molecular patterns (DAMPs) such as high-mobility group protein B1 (HMGB1) and ATP, which enhance the tumor-killing effect of CD8+ T cells by activating dendritic cells and promoting antigen presentation (42); on the other hand, oxidized phospholipids (such as oxidized PE) and lipid peroxides (LPO) released during ferroptosis can induce M2 macrophage polarization in the tumor microenvironment, promoting the expansion of regulatory T cells (Treg) by secreting immunosuppressive factors such as TGF-β and IL-10, and establishing a negative feedback mechanism for immune escape (43). Interestingly, CD8+ T cells exhibit bidirectional regulation in this process: the IFN-γ they secrete enhances tumor sensitivity to ferroptosis by downregulating the expression of SLC7A11 in tumor cells (44), but T cells themselves have increased GPX4 dependence due to their high metabolic demands and are prone to autologous ferroptosis during GPX4 inhibitor treatment. This “killing-self-destruction” effect may weaken the synergistic therapeutic effect of immune checkpoint inhibitors and ferroptosis inducers (45, 46).

## 3 Radiation-induced ferroptosis

The hallmarks of ferroptosis are the accumulation of ROS, lipid peroxidation, and a decrease in GSH levels. Numerous studies have shown that after radiotherapy, indicators of lipid peroxidation, such as ROS and malondialdehyde (MDA), are significantly upregulated, while antioxidant components such as GSH and GPX4 are significantly downregulated. These consistent findings indicate that radiotherapy can induce ferroptosis in both normal cells and tumor cells (47).The mechanism by which radiation induces ferroptosis in cells has not been fully elucidated, such as hydroxyl radicals and hydrogen peroxide. Here, we summarize several potential mechanisms involved.

### 3.1 Radiation causes excessive production of oxidized substances in cells

Radiation can directly induce the breakdown of water molecules into ROS, leading to the accumulation of intracellular ROS. Hence, radiotherapy induces the accumulation of oxidative substances within cells, leading to lipid peroxidation in cellular membranes, which disrupts membrane structural integrity and thereby triggers ferroptosis (48). The antioxidant capacity of membrane lipids against ROS varies depending on their composition; unsaturated fatty acids exhibit lower oxidative resistance compared to saturated fatty acids. Consequently, the abundance of PUFAs in cellular membranes significantly influences cellular sensitivity to ferroptosis (49). Acyl-CoA synthetase long-chain family member 4 (ACSL4) is a key enzyme in PUFA synthesis. Radiation can upregulate ACSL4 expression to promote PUFA synthesis and increase cellular sensitivity to ferroptosis. Cells lacking ACSL4 exhibit increased radioresistance (50). Research by Bach et al. revealed that knocking down Bone morphogenetic protein 4 (BMP4) in non-small cell lung cancer affects fatty acid metabolism by inhibiting ACSL4 expression (51). BMP4 is upregulated in bone marrow cells after radiation exposure (52). Therefore, one of the mechanisms by which radiation upregulates ACSL4 may involve the BMP4-ACSL4 axis.

### 3.2 Radiation reduces intracellular antioxidants

Radiotherapy can cause the depletion of antioxidants within irradiated cells, thereby inducing ferroptosis. Studies have demonstrated that intracellular GSH levels are significantly reduced following radiotherapy. In the antioxidant system, GSH plays a vital role as a reducing agent. It can reduce peroxidized polyunsaturated fatty acids through the catalysis of GPX4, thereby inhibiting ferroptosis. Radiation can inhibit the expression of SLC7A11, which affects cystine uptake and leads to GSH depletion (53). GSH suppresses ferroptosis by reducing PUFAs through the catalytic activity of GPX4. Additionally, radiation can decrease the catalytic activity of GPX4. Therefore, radiation weakens the cellular antioxidant defense system and promotes ferroptosis (54).

### 3.3 The role of the p53 protein in the regulation of ferroptosis due to radiotherapy

**Figure 1:**
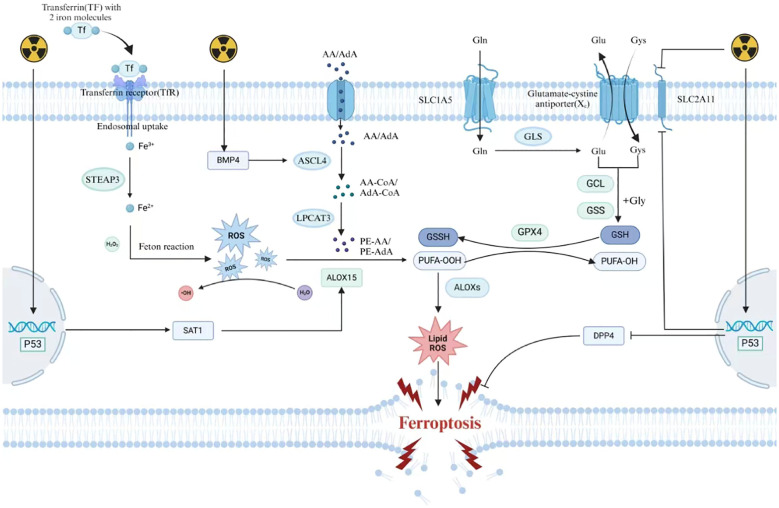
Mechanisms of radiation-induced ferroptosis.

p53 plays a critical role in regulating the ferroptosis pathway. The regulatory effect of p53 on ferroptosis is bidirectional (36). P53 has been shown to directly inhibit the expression of SLC7A11 (37) and can upregulate ALOX15 by activating spermidine/spermine N1-acetyltransferase 1 (SAT1), thereby promoting ferroptosis (38). Conversely, other studies have shown that p53 can inhibit ferroptosis by inhibiting the activity of dipeptidyl-peptidase-4 (DPP4) and suppressing its expression in a transcription-independent manner (55). As p53 is a central effector molecule in radiotherapy, it can be activated by radiation to regulate downstream signaling (56). Therefore, the activation of p53 plays an important role in IR-induced ferroptosis. Given the bidirectional regulatory role of p53 in ferroptosis, further research is needed to elucidate the potential mechanisms of p53 in radiation-induced ferroptosis (**Figure 1**).

## 4 The relationship between ferroptosis and the sensitivity of tumor cells to radiotherapy

Apoptosis is one of the important mechanisms by which radiation kills tumor cells. However, the inhibition of tumor cell apoptosis greatly reduces the sensitivity to radiotherapy and impairs its efficacy. Fortunately, although most tumor cells have developed resistance to apoptosis, they surprisingly exhibit high sensitivity to ferroptosis (5). This characteristic provides us with a new research direction: inducing tumor cell ferroptosis to compensate for defects in apoptosis resistance and thereby increase sensitivity to radiotherapy.

### 4.1 The mechanisms underlying the sensitivity of tumor cells to ferroptosis

Early researchers discovered that tumor cells exhibit high sensitivity to ferroptosis (5, 57). Subsequently, numerous studies have been conducted to explore the underlying mechanisms and identify potential targets for inducing ferroptosis in tumor cells. Investigating the mechanisms underlying tumor cell sensitivity to ferroptosis will help guide the development of clinical cancer treatments and address challenges related to radioresistance.

#### 4.1.1 PUFA content of tumor cell membranes

The expression of various PUFA synthesis enzymes is upregulated in most tumor cells. As mentioned earlier, ACSL4 is a key enzyme involved in PUFA synthesis. Research has shown that many cancers overexpress ACSL4 (15). Another crucial enzyme in PUFA synthesis is alkylglycerone phosphate synthase (AGPS), and studies have shown that the overexpression of AGPS in renal clear cell carcinoma results in elevated levels of PUFAs in cells (5). Additionally, research has shown the upregulation of other PUFA synthesis enzymes in cancer cells, such as elongation of very long-chain fatty acid protein 5 (ELOVL5) and fatty acid desaturase 1 (FADS1) (58). Compared to normal cells, tumor cells have a greater abundance of PUFAs on their cell membranes, increasing their susceptibility to oxidation by ROS and hence increasing their sensitivity to ferroptosis.

#### 4.1.2 Epithelial-mesenchymal transition

There is crosstalk between the signaling pathways associated with epithelial-mesenchymal transition (EMT) and those involved in ferroptosis. EMT refers to a process where a group of epithelial cells gradually lose their epithelial characteristics in response to specific signals and environmental stimuli, transforming into cells with mesenchymal traits. During cancer development and metastasis, tumor cells acquire enhanced invasiveness and migratory capabilities through EMT (59). Zinc finger E-box binding homeobox 1 (ZEB1) is a transcription factor activated during the EMT process that promotes the transition from an epithelial phenotype to a mesenchymal phenotype by suppressing the expression of the epithelial anchoring protein E-cadherin (CDH1). Lowering ZEB1 levels can inhibit or even reverse EMT (60, 61). Additionally, ZEB1 is a key regulatory molecule in lipid metabolism and can promote PUFA synthesis.

Tumor cells with high ZEB1 expression exhibit heightened dependence on GPX4, as GPX4 counteracts the oxidative effects of intracellular peroxides on PUFAs in cellular membranes, thereby attenuating ferroptosis. Therefore, the upregulation of ZEB1 during EMT enhances the sensitivity of tumor cells to ferroptosis (62).

#### 4.1.3 Hypoxic conditions promote ferroptosis

The hypoxic state in the tumor microenvironment can also increase the sensitivity of tumor cells to ferroptosis. Chronic hypoxic conditions can induce the production of ROS. Researchers have shown that ROS levels gradually increase with prolonged periods of chronic hypoxia in hepatocellular carcinoma (63), and similar observations have been made in breast cancer cells (64). This phenomenon may be related to the extension of the lifetime of mitochondrial electron transport chain under hypoxic conditions. Studies have shown that antimycin A, which acts on complex III, can generate more ROS by extending the lifetime of ubisemiquinone (65). Similarly, under hypoxic conditions, the lifetime of ubisemiquinone is also extended, resulting in increased ROS production (66). Hypoxia inducible factor 1α (HIF-1α) is an important transcription factor in the hypoxic tumor microenvironment. Under normoxia, HIF-1α is ubiquitinated by intracellular oxygen, leading to its degradation by the proteasome. Therefore, HIF-1α in tumor cells is stabilized only under hypoxic conditions (67). Under hypoxic conditions, HIF-1α becomes stabilized and subsequently upregulates the expression of hypoxia-inducible lipid droplet-associated protein (HILPDA) to promote PUFA synthesis (68, 69), thereby increasing cellular sensitivity to ferroptosis.

#### 4.1.4 Tumor-specific gene mutations

**Figure 2:**
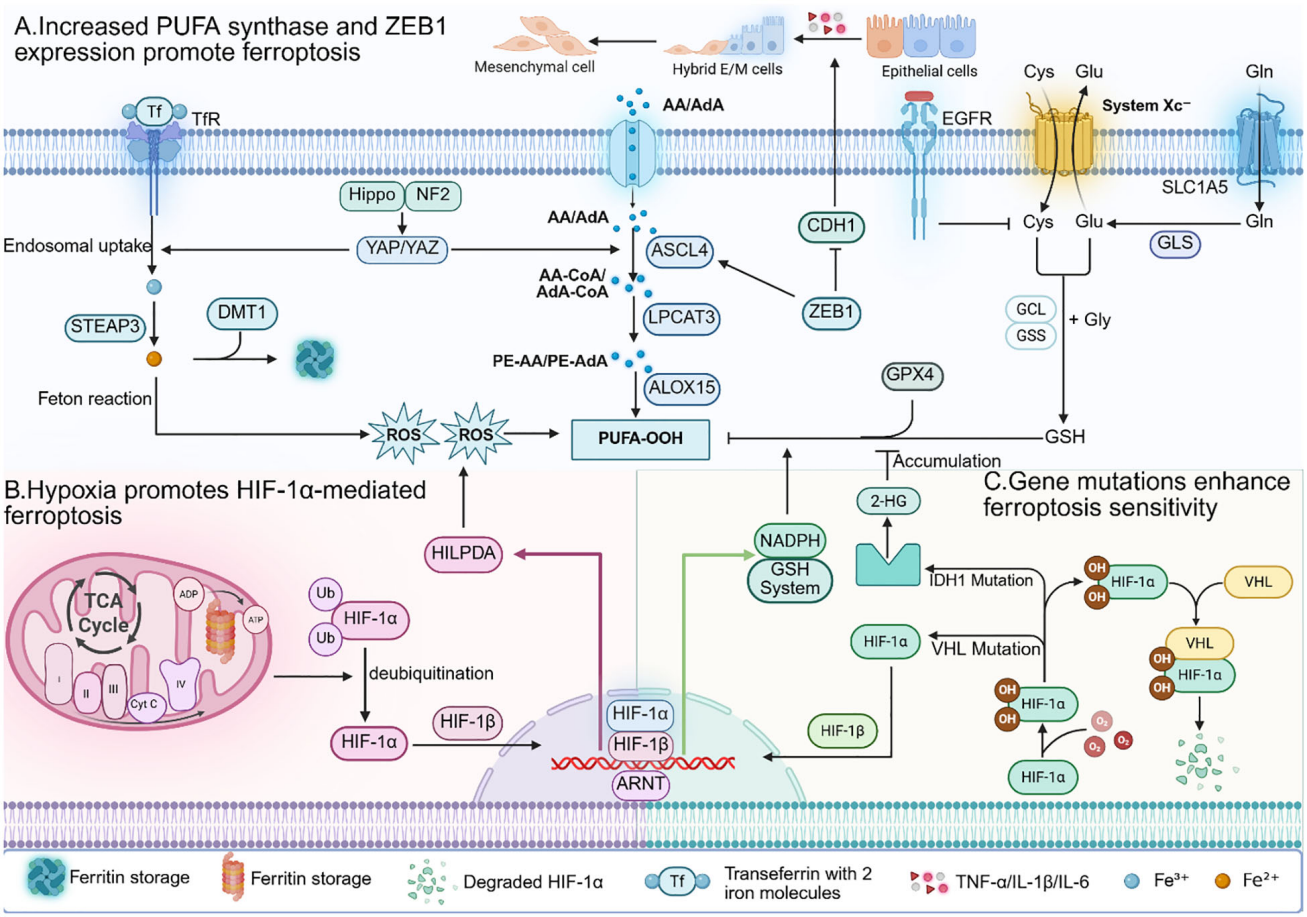
**(A)** In tumour cells, the expression of various PUFA synthesis enzymes is increased. Additionally, the transcription factor ZEB1 activated during the EMT process can promote PUFA synthesis. Therefore, tumour cells have higher levels of PUFA on their cell membranes, making them more susceptible to ROS oxidation. **(B)** Under hypoxic conditions in the tumour microenvironment, activated HIF - 1α can promote PUFA synthesis by upregulating HILPDA. However, HIF - 1α simultaneously upregulates levels of GSH and NADPH to clear intracellular ROS. **(C)** Mutations in key genes and pathways of ferroptosis often occur in tumour cells, leading to activation of ferroptotic signals and increased sensitivity of tumour cells to ferroptosis.

Tumor-specific gene mutations, activation, or overexpression confer sensitivity to ferroptosis. For example, tumor cells with mutations in the epidermal growth factor receptor (EGFR) are more dependent on cysteine, increasing their sensitivity to the inhibition of SLC7A11 and the depletion of GSH, which induces ferroptosis (70). Von Hippel–Lindau (VHL) is a tumor suppressor that is mutated or lost in most cancers (71). However, VHL is a key molecule involved in the degradation of HIF-1α and HIF-2α under normoxic conditions. It binds to oxidized HIF-1α and HIF-2α to facilitate their degradation. Loss of VHL leads to the stabilization of HIF-1α and HIF-2α, subsequently upregulating the expression of HILPDA to alter lipid metabolism, promoting PUFA synthesis (68, 69). Additionally, tumor cells with mutations in isocitrate dehydrogenase 1 (IDH1) undergo metabolic changes, leading to elevated levels of 2-hydroxyglutarate (2-HG), which inhibits the activity of GPX4, promoting the accumulation of intracellular ROS (72). Research has indicated that the E-cadherin-neurofibromin 2 (NF2)-Hippo signaling pathway exerts both tumor-suppressive and ferroptosis-suppressive effects. Many cancers exhibit inactivation of certain components in this signaling pathway (73). Mutations or inactivation of components in this pathway upregulate Yes-associated protein (YAP) and transcriptional coactivator with a PDZ-binding motif (TAZ), enhancing the expression of ACSL4, TfR1, NADPH oxidase 4 (NOX4), and other factors, thus increasing the sensitivity of tumor cells to ferroptosis (**Figure 2**) (74).

### 4.2 Inducing ferroptosis increases sensitivity to radiotherapy

As the importance of ferroptosis in tumors is increasingly recognized, ferroptosis induction in tumor cells has become a hot topic in scientific research. Many studies have focused on identifying potential targets for inducing ferroptosis in tumor cells. Here, we summarize the mechanisms that regulate ferroptosis in tumor cells and the targets of ferroptosis pathways in **Table 1**. Although these mechanisms are currently constrained in their practical applications, they offer invaluable insights for drug development. Looking ahead, drugs targeting these mechanisms could potentially be formulated in conjunction with radiotherapy, thereby enhancing tumor sensitivity and significantly bolstering the efficacy of radiotherapy. This approach holds promise for offering novel therapeutic avenues for cancer patients.

**Table 1:** Inducing ferroptosis increases sensitivity to radiotherapy.

Ferroptosis pathway targets	Cancer type	Mechanism	References
GSS	Glioblastoma	Depletion of GSS leads to disruption of GSH synthesis, leading to GPX4 inactivation and ROS accumulation, thus enhancing the induction of ferroptosis upon radiotherapy treatment.	(75)
SLC40A1	Breast cancer	iCoDMSNs (cobaltous oxide nanodots loaded into dendritic mesoporous silica nanoparticles) serve as an effective radiotherapy enhancer by upregulating HMOX1, which in turn increases transferrin receptors and decreases SLC40A1, ultimately resulting in the accumulation of Fe2+.	(76)
p53	Breast cancer	FBXW7 depletion stabilizes phosphorylated p53, inhibiting the SLC7A11-cystine-GSH axis, thereby providing an effective strategy to enhance the response of breast cancer to radiotherapy.	(77)
GSH	Breast cancer	(HMON)-GOx@MnO2 (herein referred to as PEGylated hollow mesoporous organosilica nanotheranostics) specifically releases Mn2+ by depleting glutathione, thereby activating the Fenton-like reaction, which presents a promising strategy to enhance ferroptosis induction for radiosensitization.	(78)
GSH	Breast cancer	The VGd@ICG-FA probe, which consists of hexadecyltrimethylammonium bromide (CTAB) coated with Gd2O3, loaded with ICG, and modified with FA, disrupts tetrasulfide linkages in the silica framework. This disruption leads to the depletion of GSH and the accumulation of ROS under radiotherapy, further triggering ferroptosis and enhancing the effectiveness of radiotherapy in breast cancer.	(79)
GSH	Breast cancer	BSO inhibits the biosynthesis of GSH, leading to the inactivation of GPX4. This inactivation induces robust ferroptosis, thereby enhancing the efficacy of radiotherapy.	(80)
GPX4	Human cervical carcinoma	Complex 3a (biotinylated AuI) effectively inhibits TrxR, leading to the downregulation of GPX4 and initiating the ferroptosis process, thereby sensitizing tumor cells to radiotherapy.	(81)
NRF2, GSH, GPX4	Breast cancer	AGuIX nanoparticles inhibit the NRF2-GSH-GPX4 axis, which can effectively improve the radiosensitivity of tumors	(82)
GSH	Colorectal cancer	Sulfasalazine decreases levels of glutathione and thioredoxin reductase, resulting in the accumulation of ROS. This accumulation can enhance the radiosensitivity of hypoxic colorectal cancer cells.	(83)
GPX4	Breast cancer	Tubastatin A inhibits GPX4 enzyme activity, which enhances radiotherapy-mediated antitumor effects	(84)
GPX4	Colorectal cancer	OTUD6B-AS1 overexpression stabilizes TRIM16 by binding to HuR (an RNA-binding protein) and increases GPX4-mediated ferroptosis, which improve the radiosensitivity of tumors	(85)
GPX4	Lung cancer	Heme promotes the release of GPX4 during radiotherapy, leading to an accumulation of ROS and thereby enhancing ferroptosis and radiosensitivity.	(86)
HILPDA, PLIN2	Colorectal cancer	Activation of HIF-2α upregulates lipid and iron regulatory genes, specifically hypoxia-inducible lipid droplet-associated protein (HILPDA) and perilipin 2 (PLIN2). This upregulation leads to an accumulation of ROS, which enhances the radiosensitivity of tumors.	(87)
ACSL4	Esophageal cancer	Suconazole increases the expression of ACSL4, significantly increasing lipid peroxide levels, which can increase radiosensitivity of esophageal cancer cells.	(88)
Lipid peroxidation	Esophageal squamous cell carcinoma	MF-438, an SCD1 inhibitor, reduces the synthesis of oleic acid (OA) and palmitoleic acid (POA), leading to lipid peroxidation. This process can significantly enhance radiation sensitivity.	(89)
SLC7A11	Esophageal squamous cell carcinoma	STC2 activates PRMT5, upregulating the expression of SLC7A11 and SLC3A2, which can be a therapeutic target to overcome ESCC radioresistance.	(90)
SLC7A11	Liver cancer	Low expression of COMMD10 induced by ionizing radiation inhibits the ubiquitin-mediated degradation of HIF1α. This leads to increased nuclear translocation of HIF1α and enhanced transcription of ceruloplasmin and SLC7A11, promoting ferroptosis and improving radiosensitivity.	(91)
SLC7A11	Hepatocellular carcinoma	SOCS2 promotes the ubiquitination of SLC7A11, resulting in its degradation. This process ultimately triggers ferroptosis and enhances radiosensitivity.	(92)

## 5 Ferroptosis and radiation-induced injury

### 5.1 Adverse effects of radiotherapy on non-target tissues and organs

Radiotherapy exhibits limited specificity due to its cytotoxic nature. While radiotherapy generally targets the local tumor, surrounding normal tissues and organs can also be damaged, leading to radiation-induced injury/damage (93). Radiation-induced injury is common in clinical practice and affects various organs and tissues in the body. In general, radiotherapy is more effective at killing rapidly proliferating cells. Cells in the mitotic cycle are highly sensitive to radiotherapy due to chromosomal instability (94). Rapidly proliferating cells spend more time in the mitotic cycle, increasing their susceptibility to damage from radiotherapy. On the other hand, proliferating cells have relatively weaker DNA damage repair mechanisms than quiescent cells, increasing their sensitivity to radiotherapy. Therefore, normal tissue cells with high proliferative capacity are more likely to be subject to radiation injury (95). The skin is the most commonly affected tissue, with approximately 90% of patients experiencing skin damage after radiotherapy (96). Radiation-induced lung injury is also relatively common and is primarily caused by radiation damage to proliferative type II alveolar epithelial cells (7). Additionally, radiotherapy for gastrointestinal tumors can damage intestinal epithelial cells and surrounding vascular endothelial cells, leading to radiation enteritis (97). Damage to hepatocytes can cause radiation hepatitis (98, 99). Radiation injuries to other organs and tissues, such as the urinary system, where acute nephritis and acute cystitis can occur, are also common, and in the late stages, bladder atrophy may develop (100, 101). Radiotherapy at certain sites can even damage the nervous system and the immune system (102–104). Radiation-induced injury can be categorized into acute and late effects (105). Acute radiation-induced injury occurs within three months postradiotherapy, often manifests as symptoms within one week, and is primarily characterized by intense tissue inflammation and an innate immune response (106). Late effects mainly occur after three months of irradiation and are characterized by tissue scarring and fibrosis (107).

### 5.2 Inhibiting ferroptosis reduces radiation-induced injury

The cellular mechanisms underlying the cytotoxic effects of radiotherapy are diverse and include oxidative stress, immune-inflammatory responses, cell apoptosis, necrosis, and autophagy (108). However, recent studies have highlighted the significant role of ferroptosis in radiation-induced injury. Extensive preclinical research has revealed elevated levels of ferroptosis markers in organs affected by radiation injury and has indicated that inhibition of ferroptosis in tissue cells can alleviate radiation-induced injury. Here, we summarize the findings of others to elucidate the role of ferroptosis in radiation-induced injury in various organs and tissues (**Table 2**). The basic experimental data in the table fully demonstrate the key role of ferroptosis in radiation-induced injury. Inhibiting ferroptosis can significantly reduce radiation-induced injury to tissue cells. Therefore, ferroptosis can serve as a new target for the development of radiation damage-mitigating drugs. We have compiled a list of clinically available drugs targeting ferroptosis, offering a promising strategy for mitigating radiation-induced injury.

**Table 2:** Inhibiting ferroptosis reduces radiation-induced injury.

Injury organs	Mechanism	References
Hematopoietic system, lung, and small intestine injury	Compound 5 significantly increases GSH content and upregulates the expression of GPX4 protein.	(109)
intestinal injury	EGCG reduces ROS levels and activates the transcription factor Nrf2 and its downstream targets, including antioxidant proteins SLC7A11, HO-1 and GPX4.	(110)
Intestinal injury	Radiation induces ferroptosis through the STAT1-IRF1-ACSL4 axis and causes radiation-induced intestinal injury.	(111)
Intestinal injury	TFERL inhibits oxidative stress, reduces DNA damage, and reduces apoptosis and ferroptosis, alleviating IR-induced intestinal injury.	(112)
Intestinal injury	Ferrostatin-1 alleviates radiation-induced intestinal injuries through inhibiting apoptosis and ferroptosis.	(113)
Intestinal injury	VX-765 and ferrostatin-1 alleviate radiation-induced intestinal injury through inhibiting NF-κB signaling pathway and ferroptosis.	(114)
Intestinal injury	Liproxstatin-1 inhibits ferroptosis and improves radiation-induced intestinal injury by inhibiting the LPCAT3-ALOX15 axis	(115)
Radiation colitis	Ceria nanozyme grown *in situ* on nanotubes can scavenge reactive oxygen species, and deferiprone was loaded into the lumen of nanotubes to relieve iron stress, which effectively inhibit lipid peroxidation and rescue ferroptosis in the intestinal microenvironment.	(116)
Radiation-intestinal mucositis	TBHQ inhibits 5-FU-induced ferroptosis through activating Nrf2/HO-1 pathway.	(117)
Pulmonary fibrosis	Liproxstatin-1 alleviates radiation induced-pulmonary fibrosis through downregulation of TGF-β1 by activating Nrf2 pathway.	(118)
Lung injury	Activation of the P62-Keap1-NRF2 pathway inhibits ferroptosis, alleviating radiation-induced lung injury	(119)
Lung injury	NVP-AUY922 alleviates radiation-induced lung injury by inhibiting chaperone-mediated lysosomal degradation of GPX4.	(120)
Lung injury	Radiation causes lung injury by inducing ferroptosis through downregulation of GPX4.	(121)
Lung injury	GsMTx4 reduces radiation-induced lung injury through inhibiting PIEZO1-Ca^2+^–calpain pathway and promoting the expression of GPX4 and SLC7A11.	(122)

## 6 Pharmacotherapy

### 6.1 Amifostine

Amifostine is the first clinically approved radiation protectant and reduces the impact of radiation on normal tissues by clearing ROS and inhibiting ferroptosis (123). Clinical comparative studies have shown that amifostine can significantly reduce the occurrence and severity of severe mucositis, acute or late xerostomia, and swallowing difficulties in patients with head and neck squamous cell carcinoma (HNSCC) after radiotherapy (124). Although amifostine was approved for clinical use 20 years ago, its side effects such as hypotension (125), gastrointestinal reactions like nausea and vomiting, and allergic reactions (126) have restricted its use in clinical practice (127). The typically clinically recommended dose is 200–600 mg/m², administered 30 minutes before radiotherapy and continued until the end of the radiotherapy course. For amifostine, subcutaneous injection is more helpful in reducing side effects compared to intravenous administration (128). Meanwhile, blood pressure (to prevent hypotension), liver and kidney function, and allergic reactions (such as rash and bronchospasm) must be closely monitored during medication. Although several clinical trials have demonstrated the benefit of amifostine in alleviating radiation-induced damage, its severe side effects appear to preclude providing greater benefits to some radiotherapy patients (129). A newly developed synthetic polypeptide, a glutathione analogue, reduces the toxic effects of amifostine through artificial modification while maintaining its ability to scavenge free radicals. This glutathione analogue can also synergistically alleviate radiation-induced injury by inhibiting the ferroptosis pathway (109). Compared with amifostine, this new glutathione analogue has the same therapeutic effect on radiation-induced injury and is much safer, making it a potential new radiation protectant.

### 6.2 Superoxide dismutase

Superoxide dismutase (SOD) can convert superoxide anions (O2-) into hydrogen peroxide and oxygen to remove ROS (130). By clearing accumulated ROS, SOD inhibits cell inflammation and ferroptosis, thereby exerting a protective effect against radiation injury. Several clinical studies have demonstrated the therapeutic effect of SOD on radiation-induced injury, such as GC4419 (Avasopasem, a synthetic SOD mimetic), which can alleviate mucositis in head and neck radiotherapy (131). Anderson et al. administered 90 mg/d Avasopasem via intravenous injection to patients one hour before chemoradiotherapy, and the results showed that the Avasopasem group reduced the incidence of mucositis from 67% to 42% (132). Orgotein (superoxide dismutase) can reduce the risk of cystitis and intestinal toxicity in pelvic radiotherapy patients. In a study by Esco et al., 7 weeks of treatment with Orgotein reduced the risk of cystitis by 37% and gastrointestinal toxicity by 26% (133). SOD also relieves long-term fibrosis after head and neck radiotherapy (134).

### 6.3 Melatonin

Melatonin is a powerful antioxidant that can remove ROS in the body by regulating the activity of SOD, thereby alleviating radiation-induced injury by reducing ferroptosis (135, 136). For example, melatonin can attenuate the injury by regulating classical ferroptosis pathway such as the p53 signaling pathway, the PI3K/AKT/mTOR signaling pathway, the Nrf2/HO-1 signaling pathway, and the ACSL4/CYP1B1 signaling pathway to suppress ferroptosis in these injury models (137–140). Yu et al. found that in the ultraviolet B (UVB) radiation-induced lens injury model, melatonin can regulate the activity of SIRT6 (Sirtuin 6) to modulate the SIRT6/p-Nrf2/GPX4 and SIRT6/COA4/FTH1 pathways, thereby suppressing ferroptosis and alleviating age-related changes in the mouse lens (141). In the study by Chen et al., melatonin protected hippocampal neurons from radiation-induced ferroptosis by activating the PKM2/NRF2/GPX4 signaling pathway, thereby reducing radiation-induced neuronal damage (142). Through the above-mentioned mechanisms of suppressing ferroptosis, melatonin plays a crucial role in mitigating radiation-induced injury.

The ability of melatonin to alleviate tissue organ damage caused by radiotherapy, such as mucositis, cerebral oedema, acute pneumonia, and liver damage, has been confirmed (143–146). In a Phase II clinical study, 26 breast cancer patients scheduled to receive radiotherapy were treated with melatonin cream twice daily, starting from the initiation of radiotherapy and continuing for two weeks after its completion. Results showed that the melatonin group significantly reduced the incidence of grade 1–2 acute radiation dermatitis compared to the placebo group (147). Another randomized controlled trial assigned 20 head and neck cancer (HNC) patients undergoing cranial radiotherapy to receive either 20 mg melatonin or placebo, administered daily for six weeks post-radiotherapy. The melatonin group exhibited a lower incidence and less severe severity of mucositis compared to the placebo group (148).

### 6.4 Mesenchymal stem cells

**Figure 3:**
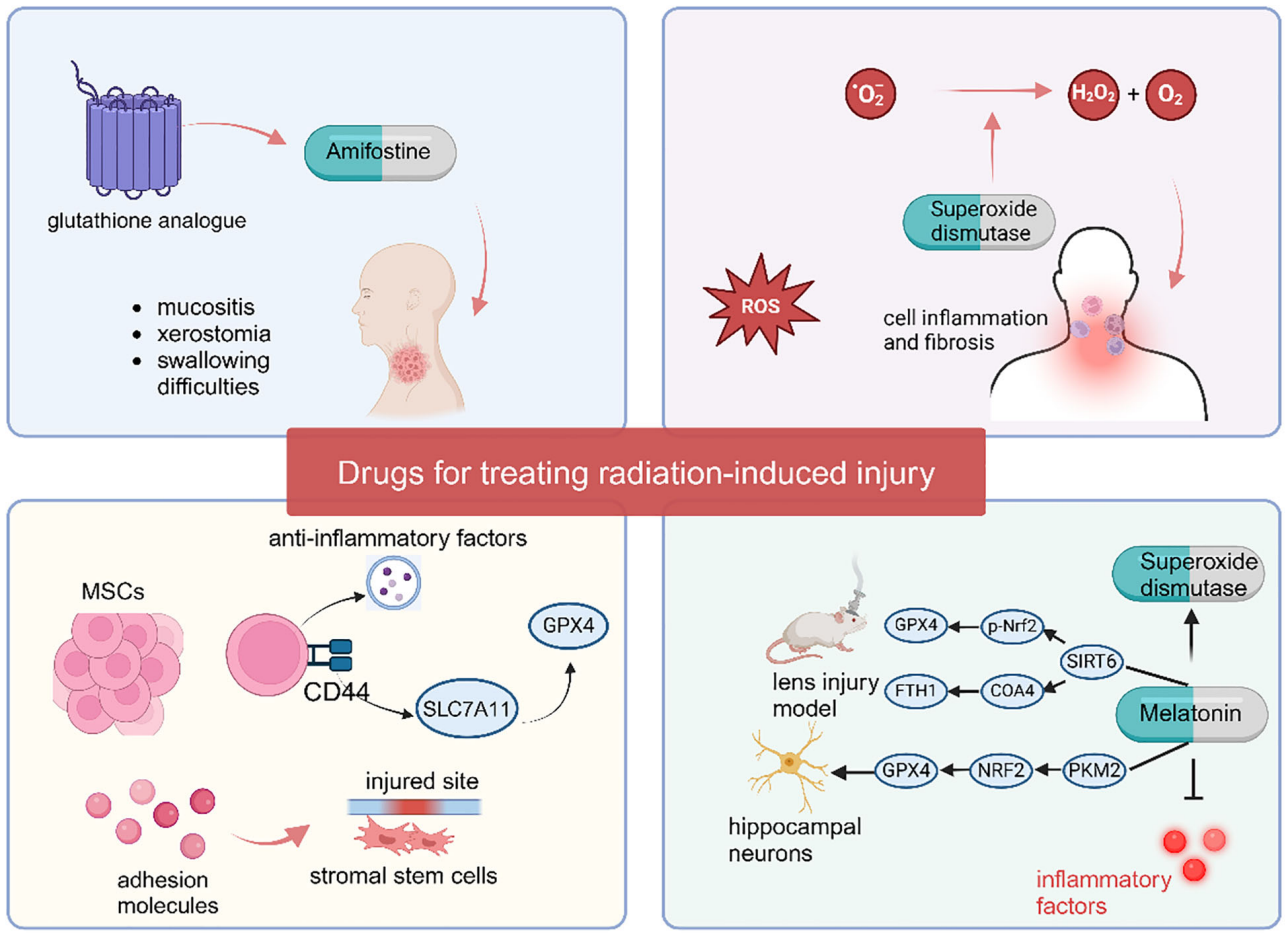
Medications targeting ferroptosis to alleviate radiation-induced injury.

Mesenchymal stem cells (MSCs) are multipotent stem cells with the potential for multidirectional differentiation. MSCs have various biological functions, among which immunosuppression and anti-inflammatory effects play major roles in preventing radiation-induced injury in cancer patients (149). When tissue or inflammation occurs, MSCs secrete various adhesion molecules to promote the homing/migration of stromal stem cells to the injured site (150). Recent study have revealed that activated mesenchymal stem cells regulate SLC7A11 expression and activity via their surface CD44 markers, directly or indirectly upregulating GPX4 protein levels, thereby reducing cellular ferroptosis susceptibility (151). Moreover, activated MSCs can secrete exosomes, releasing a large number of anti-inflammatory factors to regulate the activity of chemokines and control inflammation and immunity (152). Currently, there is a wealth of research supporting that MSCs are beneficial for preventing radiation-induced injury to various organs, such as radiation enteritis (153), radiation-induced skin damage (154), radiation-induced osteonecrosis (155), radiation-induced oral mucositis (156), and preventing long-term fibrosis (153, 157). Additionally, several clinical investigations have demonstrated that xerostomia in radiotherapy patients can be significantly ameliorated via mesenchymal stem cell (MSC) injection (158, 159). For instance, in a study by Blitzer et al., 10×10^6^ MSCs were administered via a single ultrasound-guided injection into the right submandibular gland of previously irradiated patients. No serious adverse events were observed within one month post-injection, and xerostomia symptoms were alleviated, accompanied by a substantial increase in salivary flow rate (160). However, due to the inhibitory effects on ferroptosis and immunosuppressive effects, MSCs may promote tumor cell proliferation, migration, and immune evasion. Therefore, caution is needed in the use of MSCs for tumor protection (**Figure 3**).

## 7 The contradiction of ferroptosis between radiation-induced injury and radiosensitivity

### 7.1 Ferroptosis serves as a balancing mechanism between radiosensitivity and radiation-induced injury

As previously mentioned, using these drugs to induce ferroptosis in tumor cells can effectively kill cancer cells and enhance radiosensitivity, while inhibiting ferroptosis in normal cells helps protect tissues and organs, thereby reducing radiation damage. However, the effects of these drugs lack specificity. Whether using ferroptosis inducers or inhibitors, they will circulate in the bloodstream and affect both tumor and normal cells (161, 162). Given the dual role of ferroptosis in the interplay between radiosensitivity and radiation-induced injury, either inducing or inhibiting ferroptosis can shift the balance in different directions. Therefore, it is crucial to maintain a balance between ferroptosis, radiosensitivity, and radiation-induced injury. While it may seem that improving radiosensitivity and mitigating radiation injury are at odds, ferroptosis is not the sole factor involved in radiation injury and radiosensitivity. Modulating other factors to synergize with ferroptosis may represent a feasible approach to enhancing radiosensitivity and mitigating radiation-induced injury.

### 7.2 Drugs that can both enhance radiosensitivity and reduce radiation-induced injury

Although optimal radiotherapy effects cannot be achieved solely through the ferroptosis pathway, certain drugs can not only inhibit ferroptosis to mitigate radiation damage but also enhance radiosensitivity through their selective cytotoxic effects on tumor cells. Therefore, identifying drugs that can combine ferroptosis with other tumor cell-killing mechanisms presents a potential solution to the paradox of radiotherapy sensitivity and radiation injury. Here, we summarize the following drugs in the hope of providing new insights and directions for clinical radiotherapy.

#### 7.2.1 Melatonin inhibits ferroptotic damage in normal tissues and suppresses tumor growth

Studies have shown that melatonin, in addition to inhibiting ferroptosis, also exhibits a specific killing effect on tumor cells. We hypothesize that the specific tumor cell killing effect of melatonin compensates for the protective effect of ferroptosis inhibition on tumor cells, thereby simultaneously achieving enhanced radiosensitivity and reduced radiation-induced injury. Studies have demonstrated that melatonin can regulate tumor energy metabolism and inhibit tumor glycolysis by restoring mitochondrial function through scavenging mitochondrial ROS, thereby inhibiting tumor cell proliferation (163). In addition, melatonin can downregulate vascular endothelial growth factor (VEGF) and inhibit neovascularization, thereby reducing the energy and oxygen supply to tumors (164). Second, melatonin can regulate the expression of cell cycle proteins and cyclin-dependent kinases (CDKs), causing extension of the G1 phase of tumor cell mitosis (165). It can also upregulate Bax and downregulate Bcl-2 to inhibit the progression of the cell cycle from the G1 phase to the S phase, causing tumor cells to stay in the G1 phase and increasing DNA vulnerability to damage (166). Moreover, melatonin can enhance the activity of immune cells such as NK cells and T cells, enhancing the efficacy of tumor treatment (167). Therefore, although melatonin, like most antioxidants used to treat radiation damage, protects tumor cells and facilitates radioresistance, its other tumor-inhibiting effects compensate for its protective effects on tumors.

In addition, melatonin exhibits different effects on tumor cells compared to normal cells. Endoplasmic reticulum (ER) stress occurs when cells experience environmental pressures such as hypoxia, inflammation, and oxidative stress, leading to ER dysfunction, abnormal protein folding, and the activation of various signaling pathways that affect cellular homeostasis (168). Studies have shown that in tumor cells, melatonin can induce excessive ER stress, resulting in cell damage and death. In contrast, melatonin has been found to inhibit ER stress in normal cells, thereby maintaining cellular homeostasis. This indicates that melatonin not only exerts cytotoxic effects on tumor cells but also protects normal cells from damage induced by external stressors (169). The opposing effects of melatonin on tumor and normal cells may explain why its co-administration during radiotherapy can enhance radiosensitivity while also protecting normal cells from radiation-induced injury.

#### 7.2.2 The way curcumin affects ferroptosis depends on the type of tissue

Like melatonin, some natural antioxidants, such as curcumin, have been proven to have radioprotective effects and also increase radiosensitivity. Curcumin is a natural polypeptide drug derived from turmeric that has powerful antioxidant and anti-inflammatory effects. In addition to directly scavenging peroxides, curcumin can enhance the oxygen scavenging ability by regulating the synthesis and activity of various oxidative enzymes (170). Moreover, curcumin inhibits the production of inflammatory mediators by regulating inflammatory signaling pathways such as nuclear factor κ-B (NF-κB), mitogen-activated protein kinase (MAPK), and activator protein 1 (AP-1) pathways (171). Numerous studies have shown that curcumin can induce ferroptosis in cancer cells while acting as an inhibitor of ferroptosis in tissue injury. In normal tissue injury models, curcumin alleviates normal tissue injury by inhibiting tissue ferroptosis through multiple pathways. For example, in a myocardial reperfusion injury model, curcumin inhibits ferroptosis, autophagy, and apoptosis by upregulating HES1, effectively reducing tissue damage (172). Zhai et al. demonstrated that in a Patulin (PAT)-induced renal injury model, curcumin suppresses ferroptosis-mediated renal injury via the p62/Keap1/Nrf2 signaling pathway (173). In retinopathy, curcumin inhibits ferroptosis-mediated vascular obstruction through the CXCL10/CXCR3 axis (174). Additionally, some researchers have found that in tumor tissues, curcumin can promote tumor tissue ferroptosis through classical pathways. For instance, in osteosarcoma, curcumin induces ferroptosis in tumor cells by regulating the Nrf2/GPX4 signaling pathway (175). In colorectal cancer, curcumin modulates ferroptosis via the p53 and SLC7A11/GSH/GPX4 axis (176). Furthermore, curcumin can induce ferroptosis in tumor cells through multiple signaling pathways, such as PI3K/AKT/mTOR and HCAR1/MCT1 (177–179). The anti-tumor effect of curcumin has also been recognized, as it can inhibit tumor cell proliferation and enhance anti-tumor immunity (180–182). Several clinical studies have shown that when used in combination with radiotherapy, curcumin can alleviate adverse reactions caused by radiotherapy in cancer patients and improve survival outcomes (183).

#### 7.2.3 Drugs that have both the capacity to enhance radiosensitivity and to attenuate radiation damage

**Figure 4:**
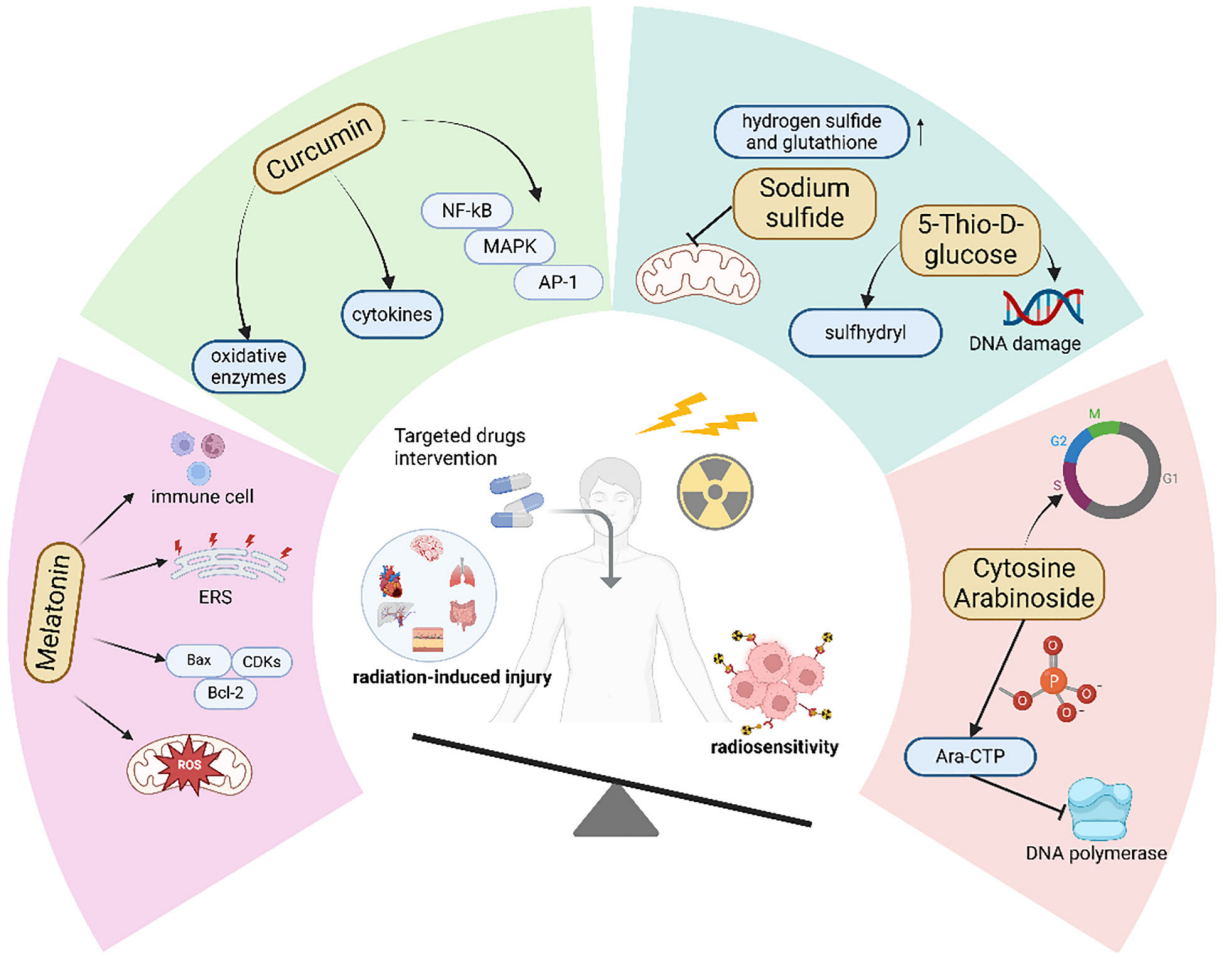
Drugs that can both enhance radiosensitivity and reduce radiation-induced injury.

In addition to the aforementioned agents, there are other drugs with similar mechanisms of action. For example, sodium sulfide (Na2S) can increase the levels of intracellular reducing substances such as hydrogen sulfide and GSH, thereby inhibiting radiation-induced oxidative stress to mitigate radiation injury (184, 185). At the same time, Na2S can also suppress the mitochondrial function and energy metabolism of tumor cells, thereby enhancing the sensitivity to radiotherapy (186). 5-Thio-D-glucose, after metabolic breakdown, releases free sulfhydryl (-SH) groups, which can lower the cellular oxidation level and thus reduce radiation damage (187), while also inducing DNA damage in tumor cells to increase radiosensitivity (188). Notably, the clinical application of sodium sulfide (Na_22_S) is constrained by its suboptimal pharmacokinetic properties. Additionally, Cytosine Arabinoside (Ara-C) is a cell cycle-specific cytotoxic agent that can cause cell cycle arrest in the S phase, which is more resistant to radiation (189). Meanwhile, Ara-C is also an effective antineoplastic drug. Upon phosphorylation by kinases, Ara-CTP can inhibit tumor cell DNA polymerase, thereby suppressing tumor growth (190) (**Figure 4**).

## 8 Radiation-induced tissue damage: individualized prevention

Radiation-induced damage is mostly related to the irradiated site, and clinical prevention and treatment of radiation injury primarily focus on post-injury management. The most common approach is treating local inflammation, such as using protective gels or short-acting low-dose hormones to control inflammation. However, existing treatments for long-term irreversible damage caused by radiation still have limitations. Both acute short-term injuries and long-term complications significantly affect patients’ quality of life (191). Therefore, establishing a personalized prevention system for radiation injury based on individual characteristics is crucial.

Current studies show significant interindividual variability in the protective effects of drug-combined radiotherapy. For instance, amifostine exhibits remarkable individual differences in efficacy and toxicity: it requires alkaline phosphatase (ALP) for conversion *in vivo* to exert its effects, and varying ALP levels among tumor patients affect the production of its active metabolites, thereby influencing therapeutic efficacy (192). Additionally, amifostine generally demonstrates better mucosal protection in head and neck cancer patients than in pelvic tumor patients (193). Pathological staging also significantly impacts the selection of protective strategies—melatonin levels are significantly reduced in tumor patients, with advanced cervical cancer patients showing notably lower melatonin levels than early-stage patients, which suppresses melatonin’s protective effects *in vivo* (194). Meanwhile, pathological types affect treatment outcomes: melatonin significantly inhibits cell growth and invasion in HER2-positive breast cancer while enhancing radiosensitivity, but this effect is less evident in triple-negative breast cancer (195).

Ferroptosis is one of the main culprits of radiation-induced normal tissue damage, and personalized treatment strategies hold greater potential in the field of ferroptosis-regulating drugs. Molecular mechanisms of ferroptosis rooted in the intricate interplay among iron metabolism, lipid peroxidation, and antioxidant systems. For example, SLC11A2 (DMT1), a divalent metal transporter, facilitates extracellular iron uptake when highly expressed; however, in specific genetic contexts, its abnormal splicing or functional inactivation may lead to insufficient intracellular iron accumulation, thereby limiting Fenton reaction efficiency and attenuating radiotherapy-induced lipid peroxidative damage (196).Conversely, overexpression of ACSL3, a member of the long-chain fatty acid-CoA ligase family, enhances the activation of polyunsaturated fatty acids (PUFAs) such as arachidonic acid (AA), promoting the synthesis of oxidation-sensitive phospholipids like AA-PE and significantly increasing membrane susceptibility to radiotherapy-induced lipid peroxidation (197).

Recent investigations have also uncovered variability in the capacity for intercellular ferroptosis propagation. For instance, Galectin-13 promotes ferroptosis spread by reducing the membrane localization of SLC7A11 in neighboring cells, a process regulated by the PKCβII-FOXK1 signaling axis with interindividual variability in activity (198). To address these mechanistic differences, researchers propose integrating real-time microenvironmental monitoring technologies (e.g., PET imaging targeting lipid peroxidation products) with multi-omics gene expression profiling to dynamically assess ferroptosis-related pathway activities. Examples include predicting monounsaturated fatty acid-mediated radioresistance risk by detecting ACSL3/4 expression levels (197, 199), and optimizing ferroptosis induction strategies for NSCLC patients using TRIM3-SLC7A11 ubiquitination degradation markers (200). These advancements provide a theoretical basis for precision radiotherapy strategies targeting interindividual ferroptosis variability, though large-scale clinical studies are still needed to validate their translational potential. Future clinical translation should establish multidimensional predictive models that integrate genomic characteristics, tumor microenvironment features, and treatment parameters to dynamically adjust protective protocols. By implementing precision medicine strategies, we can not only enhance the safety of radiotherapy but also optimize drug exposure to potentiate antitumor efficacy. This approach holds promise to maximize radiotherapy efficacy while minimizing treatment-related damage to the greatest extent.

## 9 Cutting-edge technologies in ferroptosis and radiotherapy research

The rapid advancement of biotechnology has positioned gene editing, nanotechnology, and bioinformatics as pivotal tools for unraveling ferroptosis regulatory mechanisms and optimizing radiotherapy strategies. These technologies offer innovative solutions to address the paradox of enhancing radiosensitivity while minimizing radiation-induced injury by enabling precise modulation of ferroptosis pathways, improving tumor targeting, and sparing normal tissues.

Nanocarriers have emerged as core platforms for targeted delivery of ferroptosis inducers, leveraging their tunable physicochemical properties. pH/glutathione (GSH)-responsive nanoparticles, engineered with a tannic acid-Fe²^+^ framework encapsulating sodium persulfate (Na_22_S_22_O_8_), selectively release Fe²^+^ and sulfate radicals (•SO_4_^-^) in the acidic, GSH-rich tumor microenvironment. This triggers synergistic Fenton reactions and lipid peroxidation to induce tumor-specific ferroptosis while minimizing oxidative damage to normal tissues in radiotherapy (201). Similarly, Iron-coordinated polymer nanoparticles (PCFD) further integrate doxorubicin (DOX) and cinnamaldehyde (CA) to disrupt redox homeostasis through triple mechanisms: Fe³^+^-mediated GSH depletion, DOX-induced ROS generation, and CA-enhanced oxidative stress (202). In hepatocellular carcinoma (HCC), further modification of such nanoparticles with sorafenib combined with radiotherapy enables a tri-modal attack—simultaneously inhibiting tumor proliferation, disrupting redox homeostasis, and activating ferroptosis pathways—thereby overcoming radiotherapy resistance and improving therapeutic outcomes (203). These nanocarriers enable targeted delivery of drugs to tumor cells, potently inducing ferroptosis while minimizing radiation-induced damage to surrounding normal tissues.

Integrative multi-omics analyses have become pivotal for discovering ferroptosis-related biomarkers in radiotherapy. In colorectal cancer (CRC), integrative bioinformatics screening identified METTL17 as a mitochondrial regulator whose overexpression correlates with ferroptosis resistance. METTL17 knockdown sensitizes CRC cells to radiotherapy by impairing mitochondrial RNA methylation, which disrupts mitochondrial protein synthesis and exacerbates lipid peroxidation and ROS accumulation. This positions METTL17 as a promising target for CRC radiosensitization (204). Another study revealed that phosphoserine aminotransferase 1 (PSAT1), overexpressed in CRC, suppresses ferroptosis by maintaining redox balance. Genetic or pharmacological inhibition of PSAT1 combined with radiotherapy elevates ROS, malondialdehyde (MDA), and labile iron pool levels while downregulating SLC7A11 and GPX4. Machine learning-based models, stratified by GPX4 mutation status or iron metabolism gene signatures, are being developed to predict nanodrug efficacy, advancing personalized ferroptosis-targeted therapy (205).

Gene editing technologies, particularly CRISPR-Cas9 systems, have enabled precise interrogation of anti-ferroptotic genes to enhance radiosensitivity. In breast cancer, ADAR1 (an RNA-editing enzyme) suppresses ferroptosis via the miR-335-5p/Sp1/GPX4 axis. CRISPR-mediated ADAR1 knockout downregulates GPX4, amplifying iron-dependent lipid peroxidation and restoring radiosensitivity (206). For glioblastoma (GBM), a blood-brain barrier-penetrating delivery system was developed using Angiopep-2/TAT peptide-modified extracellular vesicles (EVs) to transport Cas9/sgRNA complexes targeting glutathione synthetase (GSS). GSS ablation depletes GSH reserves, inactivates GPX4, and triggers iron overload, effectively reversing radiotherapy resistance in GBM models (75).

These cutting-edge technologies not only deepen our understanding of ferroptosis but also pave multifaceted pathways to enhance radiotherapy efficacy while reducing toxicity. By enabling targeted delivery, genetic reprogramming, and precision stratification, they mark a transition from “empirical combination therapy” to “mechanism-driven precision intervention” in cancer treatment.

## 10 Unanswered problems

Radiotherapy stands as a cornerstone in the clinical management of malignant tumors. However, its efficacy is often hampered by inherent or acquired radiation resistance in tumors. The discovery of ferroptosis has introduced a novel dimension to radiotherapy, as tumor cells tend to be highly susceptible to this form of cell death. Although ferroptosis has achieved promising results in the field of radiotherapy, radiation-induced injury should not be overlooked.

The regulatory mechanisms of ferroptosis in tumor cells remain unclear. Are tumor cells necessarily more sensitive to ferroptosis? While this review summarizes various mechanisms through which many tumors exhibit sensitivity to ferroptosis, a significant number of studies indicate that certain tumors display resistance to it. For instance, some breast cancer cells express prominin-2 to facilitate the efflux of intracellular iron (207), while also exhibiting lower levels of ACSL4 expression (15). Lung cancers with KRAS mutations are often associated with the overexpression of ACSL3, allowing these tumors to synthesize more MUFA-PL and thus resist ferroptosis (208). Additionally, lung adenocarcinoma cells show overexpression of NFS1, enabling them to store iron in ferritin and enhancing their resistance to ferroptosis (209). Consequently, the mechanisms underlying the role of ferroptosis in tumor cells are quite complex. Further research is needed to elucidate the detailed mechanisms.

There are certain contradictions regarding the topic of hypoxia and sensitivity to ferroptosis. As mentioned earlier, we indicated that under hypoxic conditions, HIF-1α can enhance ferroptosis sensitivity by promoting the synthesis of PUFAs. However, the stabilization of HIF-1α also activates downstream glutathione systems and NADPH activity to counterbalance excess ROS within the cell (65). In oral squamous cell carcinoma, HIF-1α can activate GPX4 by inhibiting the transcription of PER1, thus mediating the tumor’s resistance to ferroptosis (210). In hepatocellular carcinoma, hypoxia upregulates SLC7A11 by inhibiting the expression of methyltransferase 14 (METTL14), further suppressing ferroptosis (211). The differing effects of hypoxia on ferroptosis in tumors may explain the variations in sensitivity to ferroptosis among different types of tumors. This also highlights why overcoming the hypoxic tumor microenvironment could be an effective strategy to enhance radiosensitivity (212). The effects of hypoxia and ROS on ferroptosis sensitivity are intricate. Further research is warranted to elucidate the specific mechanisms underlying this intricate interplay.

Given the bidirectional role of ferroptosis in radiosensitivity and radiation-induced injury, rational utilization of ferroptosis-targeted approaches and strategies for striking a balance between radiotherapy efficacy and radiation-induced injury are the keys to optimizing clinical radiotherapy. However, striking this balance is still a challenge. We hope in the future, the development of specific carriers with high affinity for tumors will enable targeted delivery of drugs to tumor cells, thereby enhancing the selectivity of treatments and minimizing injury to normal tissue cells.

The main mechanisms by which drugs prevent and mitigate radiation-induced injury involve inhibiting ferroptosis, oxidative stress, and inflammatory responses. However, these mechanisms are also the main mechanisms by which radiotherapy kills cells. The use of these drugs protects both normal cells and tumor cells from radiation injury, which might lead to radioresistance. Therefore, clinicians should be cautious in the selection of drugs for the treatment of radiation-induced injury in clinical practice. In this review, we demonstrate that drugs with antiferroptotic coupled with tumor-suppressive properties, can not only mitigate radiation-induced injuries but also enhance radiotherapy sensitivity. Although these drugs still lack relevant research and their clinical applicability requires further exploration, we hope that this review can provide new ideas and directions for the selection of clinical drugs to prevent radiation-induced injury, promote the development of tumor radiotherapy, and benefit public health.

## Glossary

IR: Ionizing Radiation
TfRs: Transferrin Receptors
ROS: Reactive oxygen species
GSH: Synthesis of glutathione
GPX4: Glutathione peroxidase 4
MDA: Malondialdehyde
PUFAs: Polyunsaturated fatty acids
ALOXs: Lipoxygenases
SLC3A2: Solute carrier family 3 member 2
SLC7A11: Solute carrier family 7 member 11
AIFM2: Apoptosis-inducing factor mitochondria-associated 2
FSP1: Ferroptosis suppressor protein 1
CoQ: Ubiquinone/Coenzyme Q
CoQH₂: Ubiquinol
RTA: Radical-trapping antioxidant
DHODH: Dihydroorotate dehydrogenase
GCH1: GTP Cyclohydrolase 1
BH4: Tetrahydrobiopterin
ACSL4: Acyl-CoA synthetase long-chain family member 4
BMP4: Bone morphogenetic protein 4
SAT1: Spermidine/spermine N1-acetyltransferase 1
DPP4: Dipeptidyl-peptidase-4
AGPS: Alkylglycerone phosphate synthase
ELOVL5: Eongation of very long-chain fatty acid protein 5
FADS1: Fatty acid desaturase 1
EMT: Epithelial-mesenchymal transition
ZEB1: Zinc finger E-box binding homeobox 1
CDH1: Epithelial anchoring protein E-cadherin
HIF-1α: Hypoxia inducible factor 1α
HILPDA: Hypoxia-inducible lipid droplet-associated protein
EGFR: Epidermal growth factor receptor
VHL: Von Hippel–Lindau
IDH1: Isocitrate dehydrogenase 1
2-HG: 2-Hydroxyglutarate
NF2: Cadherin-neurofibromin 2
YAP: Yes-associated protein
TAZ: Transcriptional coactivator with a PDZ-binding motif
NOX4: NADPH oxidase 4
CTAB: Hexadecyltrimethylammonium bromide
HILPDA: Hypoxia-inducible lipid droplet-associated protein
PLIN2: Perilipin 2’OA, Oleic acid
POA: Palmitoleic acid
ATM: Ataxia telangiectasia mutated
PARP1: Poly(ADP-ribose) polymerase 1
DNA-PK: DNA-dependent protein kinase
MPF: Mitosis-promoting factor
2-DG: 2-Deoxy-D-glucose
HNSCC: Head and neck squamous cell carcinoma
SOD: Superoxide dismutase
O2-: Superoxide anions
UVB: Ultraviolet B
SIRT6: Sirtuin 6
MSCs: Mesenchymal stem cells
VEGF: Vascular endothelial growth factor
CDKs: Cyclin-dependent kinases
ER: Endoplasmic reticulum
NF-κB: Nuclear factor κ-B
AP-1: Activator protein 1
Na2S: Sodium sulfide
-SH: Sulfhydryl
Ara-C: Cytosine Arabinoside
METTL14: Methyltransferase 14
