# Supplementary material for: Ferroptosis: a double-edged sword that enhances radiation sensitivity and facilitates radiation-induced injury in tumors
Source: Front Immunol. 2025 Jul 10;16:1591172. doi: 10.3389/fimmu.2025.1591172 (PMC12287047; doi:10.3389/fimmu.2025.1591172)
Supplement: Supplementary file 1 [file Table1.docx]

| **Medicine** | **Main ID** | **Disease** | **Date of registration** | **Drug** | **Dosage** | **Treatment Course** | **Allocation** | **Phase** | **Country** |
| --- | --- | --- | --- | --- | --- | --- | --- | --- | --- |
| Amifostine | CTIS2023-508728-36-00 | Breast Cancer | 31/01/2024 | GR1014 Cutaneous Gel(4.7%,2.4%) | 0.5 mL/m^2^ | between the first RT session and 4 weeks after the last one | Randomized | Phase 2 | France |
|  | NCT03702985 | Rectal Cancer | 14/06/2018 | Amifostine | 400mg/m^2^  1 times per week | 6-8 weeks after radiotherapy | Randomized | Phase 2 | China |
|  | NCT01762514 | Nasopharyngeal cancer | 22/12/2012 | Amifostine | 400mg/d  3 times per week  5 times per week | up to 7 weeks;  3 months after radiotherapy | Randomized | Phase 2 | China |
|  | NCT01288625 | Head and Neck Cancer | 01/02/2011 | Amifostine | 500 mg sc, qod, 3 times per week | 3 months | Randomized | Phase 4 | China |
|  | NCT00601198 | Colorectal Cancer | 14/01/2008 | Amifostine | - | 12 cycles | Non-Randomized | Phase 2 | United States |
|  | EUCTR2004-004288-31-IT | Head and Neck Cancer | 14/02/2007 | Ethyol*Iv Infus 5fl polv 375mg | 375 mg | - | Non-Randomized | Phase 2 | Italy |
|  | NCT00409331 | Head and Neck Cancer | 07/12/2006 | Amifostine | 500 mg in two divided doses subcutaneously given 30-60 minutes prior to IMRT. | 6 weeks after the last dose of amifostine or RT | Non-Randomized | Phase 2 | United States |
|  | NCT00318890 | Head and Neck Cancer | 25/04/2006 | Amifostine | subcutaneous injection | 1 times per days during radiotherapy. | Non-Randomized | Phase 1-2 | United States |
|  | NCT00286611 | - | 31/01/2006 | Amifostine | - | - | Randomized | N/A | United States |
|  | NCT00274937 | Nasopharyngeal Cancer | 10/01/2006 | Amifostine Trihydrate | Given subcutaneously | 1 times per days during radiotherapy. | Non-Randomized | Phase 3 | Australia/Canada/United States |
| Melatonin | IRCT20231218060453N1 | Breast Cancer | 31/12/2023 | Melatonin | 20mg per night | from first fraction to two weeks after last radiotherapy fraction | Randomized | Phase 3 | Iran (Islamic Republic of) |
|  | ChiCTR2100042695 | Lung Cancer | 26/01/2021 | RFA and Melatonin | - | - | Non randomized | Phase 0 | China |
|  | EUCTR2018-001705-91-DK | Breast Cancer | 26/07/2018 | Melatonin Cream | 25 mg | From first fraction to last day of radiation therapy | Randomized | Phase 2 | Denmark |
|  | IRCT2016021626586N1 | Rectal Cancer | 03/03/2016 | Melatonin capsules | 20 mg /D  1 hour before radiation therapy | 5 days a week for 28 days | Not randomized | Phase 2-3 | Iran (Islamic Republic of) |
|  | NCT02430298 | Head and Neck Cancer | 28/01/2014 | Melatonin | 20 mg/ 10 ml  placebo suspension gargle for 2 minutes before radiation 15 minutes | 7 weeks | Randomized | Phase 2 | Thailand |
|  | NCT05511740 | Cervical Cancer | 11/08/2022 | Afternoon Radiation | - | - | Randomized | N/A | Indonesia |
|  | NCT03833570 | Head and Neck Cancer | 03/02/2019 | Rapid Release Capsules Melatonin | 20mg/D  30 minutes before sleeping once daily | 6 weeks | Randomized | Phase 2 | Egypt |
|  | IRCT20171122037593N1 | Healthy Person | 13/03/2019 | melatonin | 100mg  1 hour before imaging | - | Randomized | Phase 3 | Iran (Islamic Republic of) |
|  | NCT00840515 | Breast Cancer | 07/02/2009 | PraevoSkin | - | - | Randomized | N/A | Israel |
| superoxide dismutase(SOD) | ChiCTR2500096277 | Cervical Cancer | 21/01/2025 | recombinant human superoxide dismutase | vaginal douching | - | Randomized | N/A | China |
|  | EUCTR2019-002745-38-GB | Head and Neck Cancer | 23/10/2019 | GC4419 (avasopasem manganese) | 9mg/ml | - | Non-Randomized | Phase 2 | Austria/Belgium/Czech Republic/France/Germany/Netherlands/Poland/Spain/Sweden/Switzerland/United Kingdom |
|  | NCT01771991 | - | 11/01/2013 | Topical Sodermix Dismutase in the form of Sodermix (SOD) | twice daily | 12 weeks | Randomized | N/A | United States |
|  | NCT01513278 | Breast Cancer | 14/12/2011 | APN201 (recombinant human superoxide dismutase (rhSOD) encapsulated in liposomal vesicles ) | 1.6 mg/mL | Starts on the day of initiation of radiation therapy and continues until the end of radiation therapy to the whole breast | Randomized | Phase 1- 2 | Austria |
|  | NCT00618917 | Non-Small Cell Lung Cancer (NSCLC) | 06/02/2008 | Manganese Superoxide Dismutase Plasmid Liposome | 0.3/3.0/30.0 mg/15ML  Given on day 1 and 3 of each week of the experimental treatment | a total of 14 doses. | Non-Randomized | Phase 1- 2 | United States |
|  | NCT04529850 | Head and Neck Cancer | 13/08/2020 | GC4419 | 90mg ,IV | over 7 weeks | Non-Randomized | Phase 2 | Belgium/Czechia/Germany/Poland/Spain/Switzerland |
| MSCs | NCT06925607 | Pelvic  Malignant Tumors | 07/04/2025 | Mesenchymal Stem Cells (MSCs) | single dose injection (120 million cells) | - | Randomized | Phase 1 | China |
|  | ChiCTR2400094739 | Cancer | 26/12/2024 | Human placenta MSCs gel | use 1 tube (1 ×10 ^6^ cells/tube) on a 15cm^2^ wound, once a day | for 6 consecutive times | Randomized | N/A | China |
|  | ChiCTR1800019309 | Malignancy | 05/11/2018 | Human umbilical cord MSCs | intravenous infusion(1×10^8^/person) once every other week | total of three times | Randomized | Phase 1- 2 | China |
| Curcumin | IRCT20220429054699N1 | Colorectal Cancer | 19/10/ 2023 | Standard curcumin capsules | 500 mg/D | from the first day for 25-28 days | Randomized | Phase 2-3 | Iran (Islamic Republic of) |
|  | NCT05982197 | Head and Neck Cancer | 22/06/2023 | Curcumin Gel | - | - | Randomized | N/A | Iraq |
|  | IRCT20190810044500N17 | - | 13/08/ 2021 | 0.1% curcumin mouthwash | 3 times a day | 3 weeks | Randomized | Phase 2-3 | Iran (Islamic Republic of) |
|  | IRCT20181208041882N3 | Breast Cancer | 16/11/2020 | Curcumin gel 2% | 2 times a day | 4 weeks | Randomized | Phase 3 | Iran (Islamic Republic of) |
|  | TCTR20200820002 | Head and Neck Squamous Cell Carcinoma (HNSCC) | 20/08/2020 | Curcumin mouthwash | 15 ml for one minute  4 times a day | From first fraction to last day of radiation therapy for 2 weeks after completion of radiotherapy | Randomized | Phase 3 | Thailand |
|  | IRCT20200513047427N1 | Breast Cancer | 12/16/2020 | nano-curcumin Capsule | 80mg/D  after breakfast | From the first radiotherapy fraction to the last radiation therapy fraction | Randomized | Phase 3 | Iran (Islamic Republic of) |
|  | IRCT20100101002950N6 | - | 12/02/2019 | Curcumin capsule | 80 mg  2 times a day | during radiotherapy (7 weeks) | Randomized | Phase 3 | Iran (Islamic Republic of) |
|  | IRCT20180416039318N2 | Head and Neck Cancer | 23/05/2018 | Curcumin capsule | 80 mg/d | during radiotherapy (6 weeks) | Randomized | Phase 3 | Iran (Islamic Republic of) |
|  | ISRCTN13817594 | Head and Neck Squamous Cell Carcinoma (HNSCC) | 11/11/2017 | turmeric extract capsules | 500mg  3 times a day,  take after food | From the first radiotherapy fraction to the last radiation therapy fraction | Randomized | N/A | India |
|  | NCT02724618 | Prostate Cancer | 09/03/2016 | Curcumin capsule | 120mg/d | 3 days before and during radiotherapy | Randomized | Phase 2 | Iran (Islamic Republic of) |
|  | CTRI/2015/12/006413 | Oral Cancer | 04/12/2015 | Capsules Curcumin BCM95 | 1000-15000mg/d | during radiotherapy (6 weeks) | Randomized | Phase 4 | India |
|  | NCT02556632 | Breast Cancer | 23/07/2015 | Curcumin-based Gel | every 4-6 hours | first day of radiation therapy and continuing until 1 week after completion of radiation therapy | Randomized | Phase 2 | United States |
|  | NCT02300727 | - | 21/11/2014 | Curcumin (BCM-95) | 3 times a day | 4-6 weeks until mucositis is resolved | Randomized | Phase1- 2 | United States |
|  | NCT01917890 | Prostate Cancer | 03/08/2013 | BCM95 Curcumin | 6 × 500 mg | during radiotherapy (7-8 weeks) | Randomized | N/A | Iran (Islamic Republic of) |
|  | NCT01246973 | Breast Cancer | 22/11/2010 | Curcumin C3 Complex | 4 × 500 mg  3 times a day | throughout course of radiation treatments plus one week | Randomized | Phase 2-3 | United States |
|  | NCT01042938 | Breast Cancer | 04/01/2010 | Curcumin C3 Complex | 500 mg  2 times a day | during radiotherapy (4-7 weeks) | Randomized | Phase 2 | United States |
